# Dietary burden calculations relating to fish metabolism studies

**DOI:** 10.1002/jsfa.7607

**Published:** 2016-02-04

**Authors:** Christian Schlechtriem, Johannes Pucher, Britta Michalski

**Affiliations:** ^1^ Department Bioaccumulation and Animal MetabolismFraunhofer IME57392 SchmallenbergGermany; ^2^ Department of Experimental Toxicology and ZEBET, Unit Animal Husbandry, Aquaculture and Reference MaterialsFederal Institute for Risk Assessment10589 BerlinGermany; ^3^ Department of Pesticides Safety, Unit Residues and Analytical MethodsFederal Institute for Risk Assessment10589 BerlinGermany

**Keywords:** pesticide regulation, feed residues, dietary exposure, aquaculture, fish feeding, risk assessment

## Abstract

Fish farming is increasingly dependent on plant commodities as a source of feed leading to an increased risk for pesticide residues in aquaculture diets and consequently their transfer into aquaculture food products. The European pesticide regulation requires fish metabolism and fish feeding studies where residues in fish feed exceed 0.1 mg kg^−1^ of the total diet (dry weight basis) to enable the setting of appropriate maximum residue levels in fish commodities. Fish dietary burden calculation is therefore an important prerequisite to decide on further experimental testing as part of the consumer risk assessment. In this review, the different aquaculture production systems are compared with regard to their specific feeding practices and the principles of dietary burden calculation are described. © 2016 Fraunhofer‐Institut für Molekularbiologie und Angewandte Oekologie. *Journal of the Science of Food and Agriculture* published by John Wiley & Sons Ltd on behalf of Society of Chemical Industry.

## INTRODUCTION

Global aquaculture underwent a steady increase within the last three decades with an average annual production rise of 8.8%.^1^ Of the 67 million tons global aquaculture production in 2012 (excluding aquatic plants), 25 million tons were produced by marine and 42 million tons by land‐based aquaculture.^1^ Nowadays, about 520 marine, brackish water, and freshwater single species or groups of species (excluding plants and mammals) are produced in aquaculture, which differ greatly in their culture requirements ranging from planktivorous, herbivorous, and omnivorous to carnivorous feeding behaviour.^2^ To account for the respective requirements, aquaculture production techniques differ greatly with regard to the cultured species and the intensity of production. Aquaculture is classified into three intensities according to yield per area or volume of water, stocking density, quantity and quality of external feed/fertiliser inputs, degree of dependency on natural food resources, level of management/technology, financial investment and recurring costs, labour requirement, and risk of diseases or technical failure.^3^, ^4^, ^5^

In extensive aquaculture, the cultured fish species are mainly from lower trophic levels and feed solely on natural food resources (e.g. bacteria, phytoplankton, zooplankton, zoobenthos, detritus, prey fish) without substantial external inputs. In semi‐intensive aquaculture, the cultured fish species (mostly omnivorous species) are grown on a combination of natural food resources supplemented by external feeds. The quantity and quality of external feed resources are chosen to supplement the diet of fish with nutrients, which are lacking in the natural food resources.^6^ As natural food is generally rich in protein, digestible energy is the first factor limiting fish growth in semi‐intensive aquaculture and is typically supplied to fish in the form of plant‐based feeds.^7^ In intensive aquaculture, typically high‐priced carnivorous or lower‐priced omnivorous fish species are produced on large scale for national and international markets and are grown solely on pelleted, extruded, or powdered external feeds. These are formulated to supply the cultured species with all required nutrients and energy while the quantity and quality of proteins and lipids are the most important dietary quality parameters. The dietary requirements of the main species cultured in intensive aquaculture are well known and differ from species to species as well as in different stages of life.^8^

Particularly in the feeding of carnivorous species in intensive aquaculture, fish meal and fish oil are the traditional source of animal protein and fatty acids, especially omega‐3 fatty acids. Both fish meal and fish oil are mainly produced from pelagic forage/trash fish, but the amount of these fish landed worldwide fell from 30 million tons in 1994 to about 15 million tons in 2010 with large annual fluctuations in production caused by climatic circumstances, such as El Niño.^1^ The declining availability of pelagic forage/trash fisheries' products and the increasing demand for these resources for aquafeed production is causing the price on international markets to rise as well as stimulating the search for alternatives.^9^ Plant‐derived commodities are the only realistic alternative to replace fish meal and fish oil in rising demand in aquafeeds. With an increasing amount of plant material in aquaculture diets, there is an increasing risk for pesticide residues in aquaculture diets and their transfer into aquaculture products. The present study describes the principles of dietary burden calculation, which triggers investigations on nature and level of residues in fish as part of the European data requirements for pesticides according to Regulation (EU) No 283/2013.^10^

## POTENTIAL TRANSFER OF PESTICIDES FROM CONTAMINATED FEEDS TO CULTURED ORGANISMS

In extensive aquaculture systems there is no transfer of pesticide residues from feed into cultured organisms due to the absence of supplementary feeding. However, the direct treatment of water bodies or spray‐drift/run‐off/drainage after treatment along water bodies may result in fish exposure. Fish are very efficient in bioconcentrating dissolved chemicals from the surrounding medium via the gills which may lead to significant residues in fish tissues especially of lipophilic substances. However, the potential of pesticides to bioaccumulate in fish is evaluated as part of the environmental risk assessment, which is not within the scope of this publication.

Under semi‐intensive aquaculture conditions, mainly plant‐based supplemental feeds are used to supply dietary energy to the fish. These feed commodities are often of lower nutritional quality and are processed little. They range from cereals in the traditional semi‐intensive pond carp culture in Central–Eastern Europe to farm by‐products (e.g. bran) and leaves/grasses from agricultural fields in the traditional Asian poly‐carp culture.^11^, ^12^ In the global course of intensification, supplemental feeds are increasingly applied in the form of pellets. Supplemental feeding of plant‐based agricultural products and by‐products poses the risk of pesticide transfer from plants to fish and potential accumulation of residues in edible fish commodities such as fillet, liver and fat.^13^

In intensive aquaculture the fish are fed by aquafeeds which are formulated to fulfil the nutritional requirements of a particular species at its specific life stage. The feed industry uses a range of feed ingredients, which are combined according to their nutritional quality (nutrient content and digestibility) and market price in order to produce cost optimised aquafeeds with a standardised quality. The feed ingredients range from marine protein sources (e.g. fish meal, fish protein concentrates, squid meal, krill meal), processed animal protein sources (e.g. poultry meal, feather meal hydrolysates, porcine blood meal), plant protein sources (e.g. wheat and maize gluten, soybean and pea protein concentrates, soybean meal, rapeseed meal, sunflower meal), starch sources from cereals, pulses and legumes (e.g. whole wheat, wheat bran, rice bran, maize meal and bran, whole pea meal) to oils and fats (e.g. herring fish oil, anchovy fish oil, salmon oil, tuna oil, krill oil, copepod oil, soybean oil, rapeseed oil, linseed oil, palm oil, poultry fat).^14^ Some of these feed resources are processed (e.g. by heat treatment, hydrolysation) or feed additives are added in order to improve functional properties, digestibility, purity, storage properties, and stability as well as to reduce anti‐nutritional factors. Formulated feeds are either pelleted or extruded, which is a baro‐thermo‐mechanical treatment and leads to starch gelatinisation and expansion, protein denaturation, and thermal destruction of anti‐nutrients and microbial contamination. Due to the limited availability of marine protein and oil resources and the corresponding high market prices, feeds for omnivorous and even carnivorous fish species are to a large proportion plant based, which increases the risk of pesticide contaminated feeds. Pesticides may accumulate in cultured fish and potentially lead to significant residue levels in edible fish commodities. Therefore, the ingestion of pesticides and their metabolites present in fish feed has to be investigated as part of the European data requirements for pesticides both for consumer risk assessment and for the setting of appropriate maximum residue levels (MRLs) in fish commodities, which will be required as soon as individual commodities have been defined and listed for the category fish, fish products and any other marine and freshwater food products in Annex I to Regulation (EC) No 396/2005, the most recent version of which is laid down in Regulation (EC) No 752/2014.^15^

## FISH METABOLISM STUDIES

The Regulation (EU) No 283/2013^10^ sets out the data requirements for active substances in accordance with Regulation (EC) No 1107/2009^16^ concerning the placing of plant protection products on the market. Livestock metabolism studies are required where a plant protection product is to be used in crops whose parts or products, also after processing, are fed to the respective livestock species and where residues in feed may occur from the intended applications.^10^ For terrestrial livestock species this level is interpreted as residues leading to >0.004 mg kg^−1^ body weight day^−1^ (and being largely comparable to the formerly used trigger of 0.1 mg kg^−1^ feed). Livestock metabolism studies according to OECD Test Guideline 503 are established for poultry, ruminants, and pigs and are carried out to determine the qualitative and quantitative metabolism and/or degradation of the pesticide when contained in feedstuffs.^17^, ^18^ Due to the increased amount of plant derived materials in aquaculture diets, the EU Commission has placed a new data requirement also for fish metabolism data within the regulatory framework for pesticides. Fish metabolism studies need to be carried out only when the test item shows a sufficient lipophilicity (log *K*_ow_ = 3) and the dietary burden is likely to be higher than 0.1 mg kg^−1^ feed dry matter (DM).^19^ For fish no reference to body weight is made, because it changes too fast during the study (requiring adjustment of the amount of feed with time). Fish metabolism studies should be carried out according to the EU working document on ‘Nature of Residues in Fish’.^19^ Trout (*Oncorhynchus mykiss*) or common carp (*Cyprinus carpio*), which are both important aquaculture species reared for human consumption, are the recommended test species in the working document. Pilot trials with both species to study the metabolism of pesticides in farmed fish were carried out by Schlechtriem *et al.* 20 Based on the findings of the fish metabolism study and the estimated maximum residues which may occur in fish feed (maximum dietary burden), a feeding study may be required for fish,^10^ where residues at levels above 0.01 mg kg^−1^ fresh matter may be reasonably expected in edible tissues. Feeding studies are required to determine the magnitude of residues in products of fish origin in order to assess possible consumer risks arising from ingestion of these products and to establish MRLs for edible fish commodities which still need to be defined. An official test protocol for feeding studies is currently only available for terrestrial livestock species such as poultry, ruminants and pigs.^21^

## DIETARY BURDEN CALCULATION

It is anticipated that residues in fish feed which are lower than 0.1 mg kg^−1^ feed DM do not result in measurable residues in fish commodities, while this cannot be ruled out in cases of higher residues in feed. It is therefore important to calculate the level of residues in fish feed which might be reasonably expected from the approved uses of the pesticide in question. The calculation follows a conservative approach in assuming that all fish feedstuffs which could have been treated with the pesticide have in fact been treated at the maximum admissible rate. The anticipated dietary burden of a pesticide is calculated based on a ‘maximum reasonably balanced diet (MRBD)’ approach for intensive feeding practices or according to the ‘reasonably worst case diet/feed (RWCF)’ approach for semi‐intensive diets.^22^ Feedstuffs derived from field crops are defined in Annex I of the OECD guidance document on residues in livestock.^22^ Agricultural commodities and by‐products are classified into specific feedstuff categories such as forage, root and tubers, cereal grain/crop seeds, by‐products and fat. Information on the various plant commodities commonly used for the formulation of aquaculture diets and matching the categories cereal grain/crop seeds, by‐products and fat (plant/oilseed oils) is provided in Annex 2 of the EU working document.^19^

Several agricultural commodities such as forage/fodder and root and tubers, are of only limited value as ingredients for aquaculture feeds. Feed commodities can be classified as carbohydrate concentrates (CC with protein contents <30%), protein concentrates (PC with protein contents >30%), and fat (F). As described above, fish in intensive aquaculture are commonly fed based on a MRBD approach using fixed dietary levels of protein and fat. The target composition of formulated feed for rainbow trout in grow‐out culture should consist of around 42% crude protein and 15% crude fat (% of DM).^8^, ^23^ In comparison, the target composition of common carp diets contains less protein and fat with around 30–35% of DM and 5–15% of DM, respectively.^8^, ^24^, ^25^ However, no definite percentage of dietary lipids can be given for fish diets without considering the type of lipid as well as the protein and energy content of the diet.^8^

**Figure 1:**
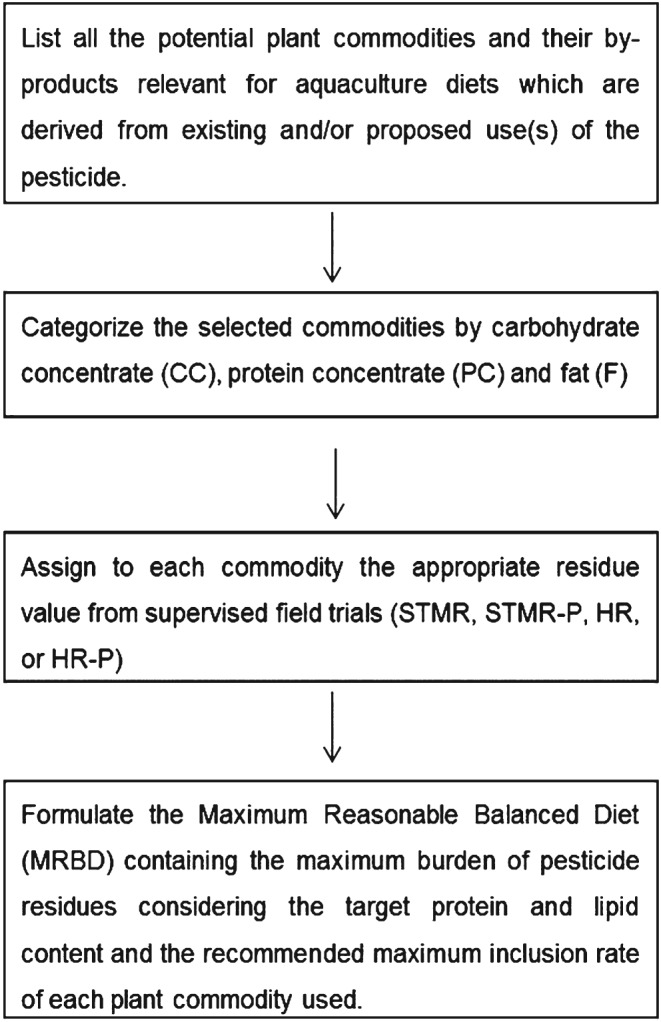
Principles of dietary burden calculation for formulated aquaculture diets.

In accordance with the calculation of residues in terrestrial livestock feed for the North American region (US/CAN),^22^ the dietary burden calculation for fish follows the principles of MRBD formulation under consideration of the appropriate residue values (mg kg^−1^ DM) for each feedstuff (Fig. 1). While for unprocessed small grains and seeds usually the supervised trial median residue (STMR) is used, for processed commodities the STMR is multiplied by a processing factor (STMR‐P) to account for an increase (processing factor >1) or decrease (processing factor <1) of residue levels during processing. If no information on processing factors is available for the particular commodity/pesticide combination, expert judgement is required to decide on a generic factor or possible extrapolations from other commodities or from related pesticides. If no processing factor can be derived, the STMR value is used together with a processing factor of 1 (assuming that residue levels do not change upon processing). Crops for which typically the highest residue value (HR) is used such as forage/fodder crops or root and tubers, or processed products for which the HR‐P would be relevant are not normally relevant for fish diets.

Recommended maximum inclusion rates are provided for the various plant commodities which should not be exceeded in rainbow trout and common carp diets to avoid adverse effects on growth performance and health condition.^26^, ^27^ The principles of dietary burden calculation for fish are described in Annex 3 of the EU working document^19^ and can be briefly summarised as follows.

First, all feedstuffs potentially treated with the pesticide are selected and the specific STMR and STMR‐P values (mg kg^−1^ DM) are listed for each feedstuff. Second, those protein concentrates (PC) and carbohydrate concentrates (CC) with the highest STMR and STMR‐P values are chosen to formulate the MRBD containing the target protein concentration of 42% or 35% of DM for rainbow trout and common carp, respectively. If the target CC or PC concentration cannot be achieved with one commodity, a further ingredient from the same group (PC or CC) is additionally considered which provides the second‐highest contribution to the overall residue and the formulation is then recalculated. Finally, the fat content of the formulated diet containing PC and CC commodities is calculated based on the lipid content of the individual ingredients. If required, the fat content of the diet is adjusted by further addition of plant derived oil (again chosen according to the highest median residue) to reach the target dietary lipid content of 15% or 10% of DM for rainbow trout and common carp, respectively. In return, PC and CC need to be aligned to maintain the target protein concentration.

Vitamin and mineral pre‐mixes are commonly applied to feed formulations to make up nutrient deficiencies. Dietary burden optimisation therefore does not consider the micro‐nutrient requirements of the target species. In specific cases, the range of commodities which are treated with a pesticide is not suitable to formulate aquaculture diets. In this case, the addition of non‐contaminated substitutes as source of PC, CC, or F might be necessary for MRBD calculation.

## DIETARY BURDEN CALCULATOR

Hand formulation of balanced diets can be carried out based on ‘Pearson's Squares’ which provide an approximation of the maximum dietary burden. However, with an increasing amount of commodities, calculations become more and more difficult and the MRBD is better calculated by linear programming to optimise the dietary burden estimates. If several feed components are available, many combinations of them could result in optimum protein and lipid concentrations for a given fish species. The software *DietaryBurdenCalculator* developed by Fraunhofer IME, Germany^28^ allows the determination of the worst case feed composition made of plant derived feedstuffs. This is the composition leading to the maximum dietary burden of pesticide residues. Calculations are based on the simplex algorithm which allows solving large‐scale linear programming problems quickly. The calculator allows estimating the specific dietary burden for common carp and rainbow trout, offers the possibility to consider maximum inclusion rates, and enables optimised residue estimation by adding non‐contaminated substitutes as a source of PC, CC, or fat (F).

## REGULATORY IMPLICATIONS OF DIETARY BURDEN ESTIMATES

Reasonable worst case estimates of the dietary burden are an important tool in a tiered pesticide risk assessment. On the one hand they provide sufficient consumer safety by applying conservative assumptions; on the other hand they prevent unnecessary animal testing by approximating realistic feeding conditions. Reasonable worst case estimates have to take into account all known uses of a pesticide which can lead to residues in feedstuffs relevant for the species under consideration (here, fish). This is not always an easy task, since the same pesticidal active substance might be contained in a variety of plant protection products with a variety of uses and information on these uses is sometimes limited. In the EU the most complete picture of feed‐relevant uses is normally obtained in the framework of evaluations of MRLs according to Art. 12 of Regulation (EC) No 396/2005.^29^ Based on the calculated dietary burden and results from metabolism/feeding studies scaled to this dietary burden, conclusions on MRLs in animal commodities are drawn. While this is currently limited to terrestrial livestock, MRL setting for fish is foreseen in future and will have to rely on appropriate dietary burden calculations for fish.

Dietary burden calculation as outlined by the EU working document^19^ is currently limited to the two recommended test species trout and carp which are considered as representatives of the full range of fish species raised for human consumption. However, the dietary burden of fish species raised in aquaculture is likely to be much more diverse. Along with the development of guidance on fish feeding studies it would therefore be desirable to develop a wider range of fish feeding tables including salt water species to derive the realistic worst case dietary burden. Testing based on this dietary burden could still be conducted with the selected representative species.

## CONCLUSIONS

Due to the increased amount of plant‐derived materials in aquaculture diets, fish might increasingly become exposed to pesticide residues through their diets. Reasonable worst case estimates of the fish dietary burden are therefore important to decide on further testing such as fish metabolism or feeding studies, which might be required to enable a reasonable consumer risk assessment and the setting of appropriate MRLs in fish commodities. A reasonably worst case diet can be obtained based on a MRBD approach for intensive feeding practices. The software *DietaryBurdenCalculator* follows a realistic dietary optimisation scenario and allows the determination of the worst case feed composition made of plant derived feedstuffs leading to the maximum dietary burden of pesticide residues.
